# Reformulating Suicide Risk Formulation: From Prediction to Prevention

**DOI:** 10.1007/s40596-015-0434-6

**Published:** 2015-12-14

**Authors:** Anthony R. Pisani, Daniel C. Murrie, Morton M. Silverman

**Affiliations:** University of Rochester, Rochester, NY USA; University of Virginia, Charlottesville, VA USA; University of Colorado-Denver, Denver, CO USA

**Keywords:** Suicide prevention, Suicide risk, Education, Assessment psychiatric services, Risk management, Educational leadership

## Abstract

Psychiatrists-in-training typically learn that assessments of suicide risk should culminate in a probability judgment expressed as “low,” “moderate,” or “high.” This way of formulating risk has predominated in psychiatric education and practice, despite little evidence for its validity, reliability, or utility. We present a model for teaching and communicating suicide risk assessments without categorical predictions. Instead, we propose risk formulations which synthesize data into four distinct judgments to directly inform intervention plans: (1) risk status (the patient’s risk relative to a specified subpopulation), (2) risk state (the patient’s risk compared to baseline or other specified time points), (3) available resources from which the patient can draw in crisis, and (4) foreseeable changes that may exacerbate risk. An example case illustrates the conceptual shift from a predictive to a preventive formulation, and we outline steps taken to implement the model in an academic psychiatry setting. Our goal is to inform educational leaders, as well as individual educators, who can together cast a prevention-oriented vision in their academic programs.

Suicidal symptoms and suicidal behavior are common among patients in psychiatric service settings, and many individuals who die by suicide have had recent contact with a mental health professional or crisis responder [1]. Educating the mental health workforce to assess and respond to suicide risk is essential to the National Strategy for Suicide Prevention [2, 3], and to efforts such as the Zero Suicide initiative for providing “suicide safer” care systems [4]. To prepare the next generation of psychiatrists for suicide prevention in behavioral health settings, training-program leadership must have a clear vision for conceptualizing and teaching suicide risk which reflects recent advances and supports prevention.

Psychiatrists-in-training typically learn that assessments of suicide risk should culminate in a probability judgment expressed as “low,” “moderate,” or “high.” This way of formulating risk has predominated in psychiatric education and practice, despite little evidence for its validity, reliability, or utility. We present a model for teaching and communicating suicide risk assessments without categorical predictions. Instead, we propose risk formulations which synthesize data into four distinct judgments to directly inform intervention plans: (1) *risk status* (the patient’s risk relative to a specified subpopulation), (2) *risk state* (the patient’s risk compared to baseline or other specified time points), (3) *available resources* from which the patient can draw in crisis, and (4) *foreseeable changes* that may exacerbate risk. An example case illustrates the conceptual shift from a predictive to a preventive formulation, and we outline steps taken to implement the model in an academic psychiatry setting. Our goal is to inform educational leaders, as well as individual educators, who can together cast a prevention-oriented vision in their academic programs. Consider the following case:

## Case Illustration: Teaching Prevention-Oriented Risk Formulation

Dr. Lang, a first-year resident, interviewed Mr. Colban and his wife in the psychiatric emergency department (ED). Mr. Colban, 54, was referred by his primary care physician, and arrived reluctantly, after endorsing “Nearly every day” on the routine depression-screening item, “Thoughts that you would be better off dead.” When his doctor asked about it, he quipped, “You never know what can happen when a guy is cleaning his gun, Doc.”

Dr. Lang determined that Mr. Colban probably had mood instability much of his life, but more erratic behavior began six months ago when he discovered his wife and his best friend in bed together. After confronting them, Mr. Colban sped off in his car and struck a concrete wall, fracturing a hip and femur. These injuries continue to cause pain.

Mrs. Colban stated emphatically that she has ended the extramarital relationship, although her husband remains suspicious, angry, and moody. He drinks with friends after work almost daily. In the heat of a recent argument, Mr. Colban said, “Maybe I should just shoot myself so you can screw Tom without guilt.” He owns a gun.

During the interview, Mr. Colban denied suicide ideation. “I say that when I’m mad, but I wouldn’t do it.” Questioned about troubling statements he made to his physician and wife, he asked, “Don’t you people have anything better to do?” Asked if he would keep himself safe he said, “Yes…I already said I would never do it.” He agreed to let a family member keep his gun temporarily.

For psychiatrists and other clinicians, arriving at a clear formulation of a patient’s level of risk, based on a synthesis of clinical information, is a core competency for assessing and managing suicide risk [5]. But, there is no clear consensus about what “risk formulation” entails [6], despite significant advances in the clinical literature on assessing and managing suicide risk. In our experience, the most common usage of the term *risk formulation* is illustrated by Dr. Lang’s assessment of Mr. Colban’s risk for suicide, as seen in the continuation of the case illustration:

After discussion with the patient, his wife, and his primary care physician, Dr. Lang reported his findings to his preceptor, Dr. Santis: “We have no grounds to keep this guy. I’m worried he might kill himself *someday*, but I don’t feel there is an immediate risk.”

Dr. Santis: “What is your risk formulation and plan?”

Dr. Lang: “Risk is low or moderate… low-moderate, I guess. He’s not reporting acute distress.”

Dr. Santis: “And your prevention plan?”

Dr. Lang: “Discharge to home, outpatient intake this week, and give them the crisis phone numbers.”

Dr. Santis: “Good start, but you’re worried about future risk. How does your formulation and plan address that?”

Dr. Lang presented his formulation as a categorical probability judgment. Such assignments of risk level are usually expressed on some type of Likert scale from low to moderate to high, often with additional gradations such as “low-moderate.”

Educational leadership requires challenging outdated paradigms, and the practice of applying simple labels to risk severity is fraught with problems: These categorical labels have poor predictive validity, inter-rater reliability, and clinical utility [7–9]. Furthermore, categorical labels tends to be ambiguous: Does “high risk” mean a patient is genuinely more-likely-than-not to die by suicide (in which case, intense and urgent intervention is warranted), or only that the patient is at higher risk than the general population (in which urgent intervention may be unnecessary)? Better alternatives involve distinguishing between long-term and short-term risk [10] or increasing specificity between different risk levels [7, 11]. Although these alternatives are clear improvements, none of them presents a comprehensive model suitable for supervision and teaching, communication among professionals and with patients, and documentation. Seeking to build on recent advances, we identified the following criteria that a practical model must meet:

1. Risk formulation should be anchored in the clinical context and patient population in which the assessment occurs [12]. Rates and risk of suicide differ across contexts [13], so clinicians in different practice contexts (e.g., outpatient, inpatient, and emergency services) will have a different experience base with distressed patients and hence different judgments about risk. A patient considered high risk in one context (e.g., a college counseling center) might be considered low risk in another context (e.g., an inpatient psychiatric hospital). These risk appraisals differ, not only because patient populations differ but also because each setting has different resources available for intervention. Likewise, the purpose of an assessment varies by setting. So, clinicians must conceptualize and describe risk in relative terms. Describing a patient as “low risk” or “high risk” in the abstract is far less meaningful than describing the patient as at lower or higher risk *relative to other patients in the same context*.
2. Risk formulation should capture the fluid nature of suicide risk in the life of an individual patient [10, 14, 15] and explicitly state: (a) how the person’s current risk compares to risk at previous time points, and (b) how risk might change in response to future events.
3. Risk formulation should lead directly to intervention strategies [16]. Data points included should provide the building blocks needed to produce risk management plans.

In this article, we propose to both broaden and refine the definition, practice, and teaching of suicide risk formulation by presenting a model that meets these criteria. Clinical and educational leaders can use this model to prepare preceptors and improve educational experiences of trainees. While clinical judgment is involved in any assessment, our model is intended to provide structure and transparency, enabling clearer communication and support for clinical decisions. We build upon recent advances in the clinical literature on assessing and teaching suicide risk [5, 7, 10, 11, 16, 17] and inform educational leaders about how to fill a gap in contemporary psychiatric education. We also provide an illustration of implementing this model in one academic psychiatry setting.

## Prevention-Oriented Risk Formulation

We define risk formulation as *a concise synthesis of empirically based suicide risk information regarding a patient*’*s immediate distress and resources at a specific time and place*. The goal of this synthesis is not to predict behavior but to promote communication and collaboration among professionals, patients, and families to reduce risk in the short and long term.

In light of this definition, we see that Dr. Lang’s view of the scope and purpose of risk formulation is too narrow. His statements indicate that risk formulation is solely a prediction of how likely Mr. Colban is to attempt suicide in the near future. Further, his initial statement to Dr. Santis, “We have no grounds to keep this guy,” reveals the common tendency to “back in” to risk formulation. In other words, he decided between one of two intervention options (release or hospitalize) and then assigned a categorical label post hoc to justify his decision. Dr. Lang’s statement also illustrates the common misconception that risk formulation is complete once immediate disposition has been determined. We emphasize that risk formulation should not be a categorical label conveying a *prediction*, but rather a synthesis of information that facilitates *prevention*.

**Fig. 1:**
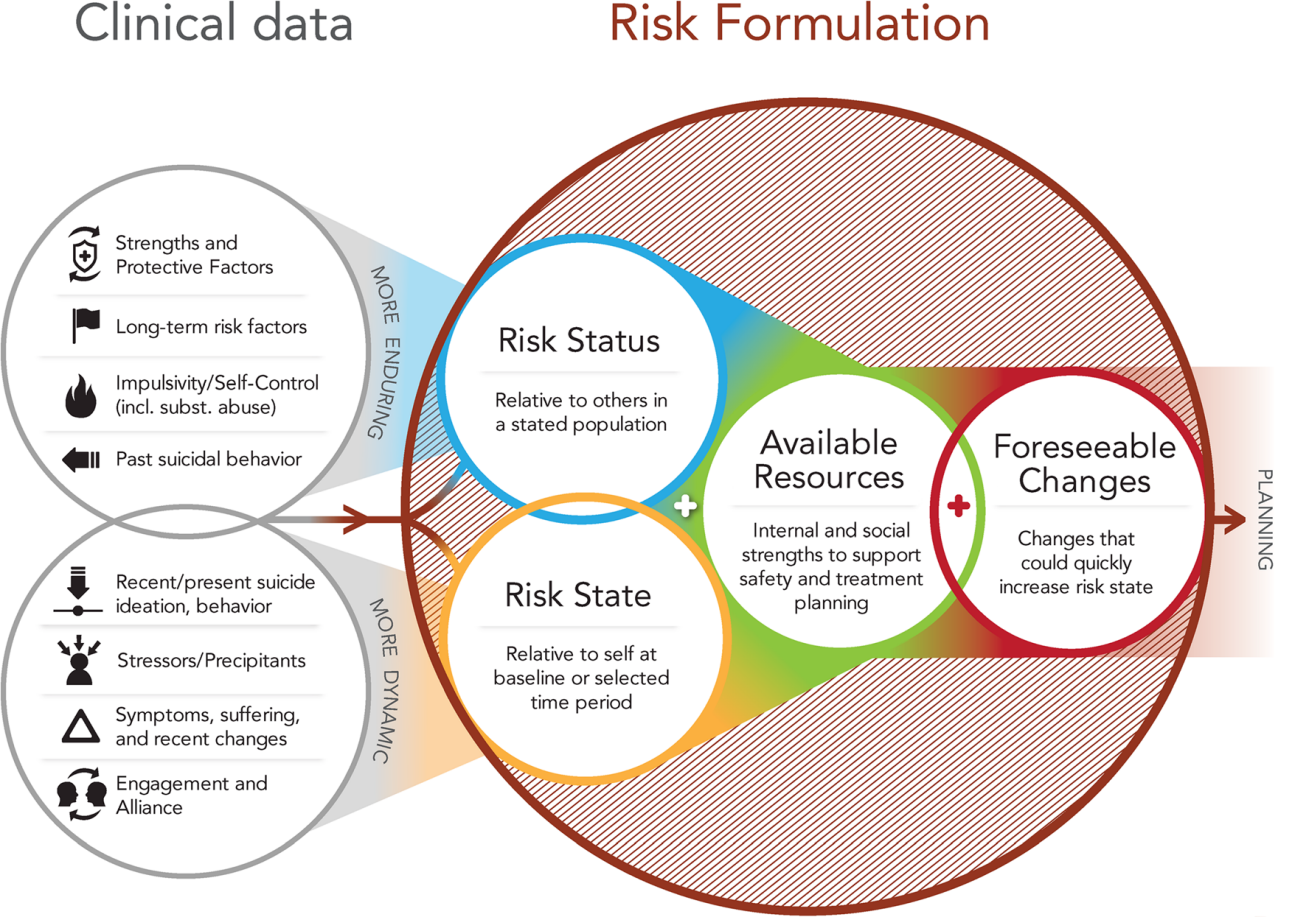
Prevention-oriented risk formulation

To broaden Dr. Lang’s view of risk formulation, Dr. Santis introduced him to a prevention-oriented model. Figure 1 diagrams a risk formulation model we use for teaching purposes. The model flows from left to right. The left side of the model shows eight domains of “clinical data” that a clinician gathers and synthesizes in collaboration with the patient and other individuals central to the patient’s life and care. These domains are adapted from those proposed and explicated by Bryan and Rudd [17] to inform risk assessment: strengths and protective factors; long-term risk factors; impulsivity/self-control; past suicidal behavior; recent/present suicidal ideation and behavior; stressors/precipitants; symptoms, suffering, and recent changes; and reliability and engagement. To highlight the importance of considering both historical background and immediate clinical presentation, the eight domains are organized into circles of “more enduring” and “more dynamic” factors. Consideration of these domains yields a judgment about risk status, risk state, immediately available resources, and foreseeable changes.

### Risk Status and Risk State

Our model for risk formulation draws on contributions from the violence prevention literature. In order to model the fluid nature of violence risk assessment, Douglas and Skeem [18] distinguished between risk status and risk state. As applied to suicide, risk status is a person’s risk of suicidal behavior relative to others in a stated population. Risk status is informed by base rates of suicide in particular populations, and well-known, empirically supported risk factors for suicide drawn from epidemiological research. These factors tend to be more enduring (i.e., fixed, historical, and static), such as history of psychiatric illness, family history of suicide, history of abuse, and history of suicidal behavior. For example, a patient with multiple suicide attempts would likely have a higher risk status than a patient with a similar diagnosis, level of current distress, or current severity of suicidal thoughts [19]. Risk state refers to a person’s current risk compared with his/her own risk at baseline or at another set point in time. A patient’s recent suicidal statements and behavior, current symptoms and stressors, and degree of engagement with helping resources all inform risk state. The factors that inform risk state tend to be more dynamic and malleable and relate more to moment-to-moment clinical status. Together, risk status and risk state yield descriptions of an individual’s current vulnerability and volatility, anchored in population, context, and time. Risk status is expressed in relative terms (“higher than,” “similar to,” or “lower than”) in relation to a relevant comparison group, as illustrated in the following dialogue between the faculty preceptor and resident in our illustrative example.

### Teaching Risk Status

Dr. Santis: “Could you indicate Mr. Colban’s risk compared with the general population?”

Dr. Lang: “I would say higher. He’s had a past suicide attempts, and some ongoing depression.”

Dr. Santis: “I agree. How about compared with other depressed patients in our outpatient service?”

Dr. Lang: “Probably middle of the road.”

Dr. Santis: “Yes, and how about compared with the last ten patients we admitted to the inpatient service?”

Dr. Lang: “A lot lower—there’s no psychosis or intoxication, and even though he gave us a hard time, he cooperated with a plan to have his brother-in-law secure his firearm and stated that he doesn’t plan to kill himself.”

Dr. Santis: “OK. Then we can say that his risk status is higher than the general population, similar to outpatients in our behavioral health service, and lower than patients typically admitted to our service.”

In prediction-based models, clinicians must integrate complex risk information to estimate the likelihood of a statistically rare event. But, there are no known algorithms for precisely weighing risk factors in individual cases, so clinicians perform poorly at the task [20]. Of course, describing risk status also requires some subjective clinical judgment and therefore remains vulnerable to bias and error. But, this task involves estimating whether an individual’s risk is similar, lower, or higher than a comparison group, a task that is simpler (and vulnerable to fewer biases) than a predictive task, or a task that involves estimating risk in the abstract. In our experience, anchoring risk to a specific relevant population enhances and clarifies communication, thereby improving the reliability of judgments across clinicians—but we emphasize that it still needs to be tested empirically. Clinicians may choose to compare risk to various comparison groups (as did Drs. Lang and Santis above), but for clinicians unfamiliar with multiple contexts, even a comparison to the current context is more precise, and therefore meaningful, than trying to describe risk in the abstract.

Risk state is expressed in terms relative to a strategically chosen point or points in the patient’s own history. Thus, the risk formulation focuses the clinician on temporal changes and how the immediate distress fits within the events and patterns of the patient’s life, as illustrated in the continuation of the case illustration.

### Teaching Risk State

Dr. Santis: “What do we know about the patient’s risk state today compared with other times in his life?”

Dr. Lang: “Well, it is obviously higher than it was before he found his wife in bed with his best friend, but none of the information we gathered indicates that his risk is higher today than it has been for the past six months. What happened today is that the PCP’s routine screen detected his risk.”

Dr. Santis: “Precisely! So we can say that his risk state is higher than his pre-morbid baseline but similar to what it has been in recent months.”

To be clear, the goal of assessing risk state is not to *predict* whether an individual will take his own life. Indeed, assessing the patient’s “worst point” (a static risk factor closely tied to past suicidal ideation and attempts) would probably be a better strategy if the goal were incremental improvement of long-term *predictions* [21]. However, the goal of assessing current risk state is not improving long-term prediction, but gauging the intervention necessary to reduce suicide risk. Comparing current risk state to the patient’s “baseline” state and worst-point state may shed light on effective interventions, and foreseeable changes that could increase or decrease risk.

### Available Resources and Foreseeable Changes

A “risk formulation” which includes only a categorical label does little to enhance prevention. This type of labeling tends to encourage little or no individualized intervention for those labeled low risk, and intense, but rarely individualized, intervention for those labeled high risk (e.g., civil commitment). Categorical labels cannot convey the detailed information necessary to tailor a risk management plan. Better risk formulation explicitly addresses the patient’s available resources and foreseeable changes crucial to individualized prevention. Available resources are those immediately accessible to the patient and treatment team to support crisis and treatment planning. They are distinguished from protective factors, which generally refer to broad strengths or epidemiologically derived variables known to decrease risk across populations, such as demographic factors, having children in the home, or holding attitudes against suicide. Protective factors are important to note but are not always immediately available to aid in a crisis.

Foreseeable changes are events or stressors, which, if they occurred, could reasonably be expected to increase or decrease risk. Identifying these potential changes as a core element in risk formulation (a) explicitly acknowledges the fluid and inherently unpredictable nature of suicide risk [10, 14, 15], and (b) directly suggests situations around which specific contingency plans can be developed in collaboration with patients and their families. Thus, the goal of anticipating changes that could increase risk is prevention, not prediction.

We suggest that clinicians try to identify at least two significant potential changes. Ideally, changes that could increase risk are ascertained in collaboration with the patient and/or others involved in the patient’s life or care. In addition, clinicians can deduce the types of changes or losses that would be particularly devastating or destabilizing based on past history and precipitants (e.g., substance use and school disciplinary action), and well-known challenging transitions (e.g., inpatient discharge), as well as an empathic understanding of the unique strengths, relationships, and activities that give meaning to the patient’s life. The clinician’s role in identifying resources must increase when a patient’s impaired mental status, insight, or cooperation reduces collaboration.

Available resources and foreseeable changes inform immediate decisions: If foreseeable changes are likely and severe, and available resources are few, the patient may require more intensive intervention. The continuation of the faculty-resident dialogue illustrates the application of these concepts.

### Identifying Available Resources and Foreseeable Changes

Dr. Santis: “To summarize Mr. Colban’s risk and make systematic prevention plans, we need to consider what events or stressors could rapidly change the situation we see now. We also need to consider what resources he and his support system can call upon if a crisis does occur.”

Dr. Lang: “He has his wife. She’s here with him and seems to be supportive, even though she’s a stressor too.”

Dr. Santis: “OK, that’s one. It’s common that an intimate partner might be both a resource or a stressor, depending upon behavior–that’s just reality. What else? When we’re discharging someone we like to name at least two solid resources. If we can’t, that’s a sign we might need to reconsider.”

Dr. Lang: “He trusts his regular doctor and goes there pretty often. That’s the person he disclosed to initially. We can see if the PCP will act as another set of eyes.”

Dr. Santis: “Great. Now, what changes could happen in Mr. Colban’s life that might rapidly escalate his risk state?”

Dr. Lang: “If he finds out his wife is still cheating…or if she leaves him.”

Dr. Santis: “Exactly! Another crisis with his wife is certainly my biggest concern.”

Dr. Lang: “And if he starts talking about shooting himself at a time when he is intoxicated, I would worry.”

Dr. Santis: “Makes sense. Then he and his wife should leave here with a specific contingency plan that addresses each of those foreseeable changes, and we’ll communicate those to the outpatient team as well.”

### Documentating Risk Formulation

Dr. Lang’s documentation reflected the systematic approach to risk formulation that Dr. Santis modeled in their case discussion. Here is an excerpt from the visit summary note entered into the record:

Formulation of Risk (Summary): Mr. Colban’s risk status is higher than the general population, but lower than patients typically admitted to the inpatient service. He is under a great deal of stress, struggles with depressed mood, and drinks regularly, but is not acutely distressed, faces no new stressors today, has had no consequences from his drinking, and has been cooperative with the planning process. Risk status is similar to that of depressed patients in our outpatient service. His current risk state is similar to his risk state throughout the previous eight months, though higher than his historical baseline. A goal for outpatient therapy would be to return to his baseline risk state. Mr. Colban’s wife and PCP, whom he sees regularly for pain management, are important available resources for him. However, should his wife leave him or new suspicions of infidelity arise, risk could increase rapidly. Likewise, Mr. Colban’s risk state could increase rapidly if he begins to contemplate suicide while under the influence of alcohol. Our team has made contingency plans for each of these foreseeable changes.

This excerpt was followed by a description of plans made with Mr. Colban, his wife, and his primary care physician with whom the team communicated during the ED visit. These plans flowed logically from the formulation. The team developed contingency plans for the foreseeable changes identified and followed a Safety Planning protocol [22] to assure the patient and support system identified other coping resources and 24-hour crisis response options. Finally, Dr. Lang documented his extensive consultation with the attending physician and the rest of the interdisciplinary team in arriving at his conclusions and recommendations. These included a recommended time frame for when the next routine follow-up assessment should occur, in addition to any that might be triggered by observed changes. Subsequent to discharge from acute services, the outpatient team used Dr. Lang’s formulation to anticipate and avert increases in risk state, and to construct long-term treatment plans to address both dynamic and, especially, enduring risk factors which could ultimately reduce risk status.

## Prevention-Oriented Risk Formulation in Academic Psychiatry: Leading the Paradigm Shift

Educational leadership often requires casting and executing a vision for new clinical paradigms in our training programs. The model articulated in this article has gained traction nationally through its adoption by existing training programs. The model has been used to train psychiatrists, psychologists, and social workers in a range of facilities, in a government-sponsored national webinar, and it has been recently adopted by two curricula disseminated nationally [23, 24]. We have adopted this model in our academic psychiatry programs at the University of Rochester, integrating it into clinical workflows, case discussions, change-of-shift reports, patient education, and documentation used in the Comprehensive Psychiatric Emergency Department for training psychiatric residents and fellows. Adopting this model required a paradigm shift for many of our faculty and staff, since most were accustomed to prediction-oriented risk categories and labels.

To shift thinking and change practices in our setting, the department chair convened a multidisciplinary leadership team to handle the educational and administrative rollout. The team consisted of a Comprehensive Psychiatric Emergency Department (CPEP) medical director, a nurse manager, a lead social worker, an electronic medical record coordinator, and a suicide prevention expert (ARP). Understanding that broad leadership support is critical for educational innovation [25], this team met with educational and clinical leaders to ensure full support of the executive team, education committee, and quality assurance before disseminating the model to faculty and staff. The educational rollout used 20 min of video-based training and 30 min of in-person training for all CPEP clinical staff. The video portion explicated the risk formulation model (shown in Fig. 1), while the in-person session walked trainees through two practice applications, modeling an adolescent and an adult patient. An introductory video for faculty and staff can be viewed at https://vimeo.com/105130731. Residency faculty and attending psychiatrists participated in an additional 30 min of in-person education, addressing common questions and special considerations for incorporating the model into resident supervision.

A poster campaign was held parallel to the rollout to familiarize staff with the model and terms used. Posters were hung at nurses’ station and other staff areas. The risk formulation model, a model for responding to identified risk, and screenshots of relevant sections of the electronic record were displayed. Pens and markers hung next to the posters, with an invitation to faculty and staff to mark screenshots with ideas, problems, and feedback. Thirty days after the initial rollout, the leadership team met again with attending psychiatrists and other interested staff to review feedback and progress and suggest future improvements.

## Discussion

National attention has focused on “suicide safer” care in behavioral health. Academic psychiatry is in the best position to lead the way toward clinical paradigms of suicide risk that change the focus from prediction to prevention. In the model we propose, the risk formulation process comprises four components flowing logically from one to the next: risk status, risk state, available resources, and foreseeable changes. This model synthesizes advances made over the past decade in suicide risk assessment [7, 10, 11, 17] with innovations in forensic assessment of violence risk [18]. In this model, assessment and description of risk are explicitly anchored in the clinical context and patient population, in the patient’s own history, and in the patient-specific opportunities for prevention. The model is straightforward, easy to remember, and suitable for teaching and supervision, communication among professionals and with patients, and documentation. The visual representation or “map” helps reinforce the relationship between constructs—a strategy consistent with research in health sciences education and best practices for cognitive schema formation and key concept retention [26].

A key strength of this model as a tool for education and practice is that it redirects clinicians’ attention away from prediction-oriented, categorical labeling and focuses on contextually anchored, prevention-oriented judgments. These judgments then directly inform person-specific plans and interventions. For example, identifying foreseeable changes provides an obvious starting point for planning: i.e., specific safety plans for each change that occurs. When teaching risk formulation, clinicians must be cognizant at every step that assessment should lead to actionable responses. Giving trainees a sense that they will develop “assessments that matter” is likely to motivate both initial learning and eventual implementation or adoption. Thus, our model focuses on prevention of future suicidal behavior, rather than prediction, and signals “forward movement” from gathering relevant data, through elements in a risk formulation that directly lead to practical safety and crisis response plans, which are key to suicide prevention in clinical settings [22].

As with all models, ours will require ongoing study and evaluation; this article provides the conceptual background for such work. Examining the impact that prevention-oriented risk formulation has on decisions, plans, and patient outcomes is an important future direction for this model and for the field of suicide prevention education [27]. We have received positive feedback from participants about the ease and utility of the model; however, empirical study of the educational value is still needed. Key questions for future study include assessing the impact of this model on the following: clinician satisfaction and self-efficacy, cross-clinician reliability in risk formulations, documentation quality, efficiency and effectiveness of team communication, patient satisfaction and perceived collaboration, and the specificity and perceived helpfulness of safety plans and dispositions.
